# Reliability-Guided Adaptive Feature Fusion Network for Noise-Robust Bearing Fault Diagnosis

**DOI:** 10.3390/s26113288

**Published:** 2026-05-22

**Authors:** Song Yang, Mei Liu, Yukang Chen, Jianfeng Zhang, Peng Wang, Pengfei Luo

**Affiliations:** 1 School of Automation, Guangdong University of Petrochemical Technology, Maoming 525000, China; yang3185113@163.com (S.Y.); 17771032603@163.com (Y.C.); 13432771905@163.com (J.Z.); 2 Guangdong Provincial Key Laboratory of Intelligent Safety for Petrochemical Equipment, Guangdong University of Petrochemical Technology, Maoming 525000, China; 17852581803@163.com (P.W.); luopengfei0506@163.com (P.L.); 3 College of Electrical and Control Engineering, Jilin University of Chemical Technology, Jilin 132022, China

**Keywords:** fault diagnosis, cross-noise, adaptive feature fusion, reliability estimation

## Abstract

Cross-noise fault diagnosis remains challenging due to the mismatch between training and testing noise conditions, which degrades feature reliability and model generalization. To address this issue, this paper proposes a reliability-guided adaptive feature fusion framework (RGAF-Net). The method focuses on sample-wise adaptive feature fusion, where the enhanced wide first-layer convolutional neural network(WDCNN) backbone is employed to improve multi-scale feature extraction under noisy environments. In addition, a dual-path architecture is introduced to provide complementary representations, including globally robust structural representations and locally detail-sensitive structural responses. Furthermore, a lightweight reliability estimation module is designed to characterize the signal degradation tendency under noisy conditions of each input sample, based on which a sample-wise routing mechanism dynamically adjusts feature contributions during feature fusion. Experiments on two public bearing datasets (PU and JNU) under cross-noise settings demonstrate that the proposed method achieves improved performance compared with representative approaches, particularly under severe noise conditions. For example, on the JNU dataset at −10 dB, the proposed method improves the Macro-F1 score by over 19 percentage points compared with the baseline WDCNN. Ablation studies and visualization analyses further demonstrate the effectiveness and adaptive fusion behavior of the proposed framework. The results indicate that the proposed method provides an effective solution for robust fault diagnosis under noise mismatch scenarios.

## 1. Introduction

Rotating machinery is a critical component in modern industrial systems, where rolling element bearings are among the most failure-prone components. Bearing failures may lead to severe economic losses and safety risks if not detected in time [1]. Therefore, accurate and reliable fault diagnosis has become an important research topic in intelligent maintenance systems. Traditional approaches mainly rely on signal processing techniques and handcrafted feature extraction. For example, Lei et al. [2] reviewed the application of empirical mode decomposition in rotating machinery fault diagnosis. Although these methods are effective under controlled conditions, they often suffer from limited generalization ability in complex industrial environments with strong noise interference and varying operating conditions.

With the rapid development of artificial intelligence, deep learning has been increasingly introduced into fault diagnosis tasks. LeCun et al. [3] summarized the fundamental framework of deep learning, and Zhao et al. [4] further reviewed its applications in machine health monitoring. Compared with traditional methods, deep learning models can automatically learn hierarchical representations from raw data, thereby reducing the dependence on manual feature engineering and improving representation capability under complex conditions.

Among various deep learning models, convolutional neural networks (CNNs) have been widely applied in fault diagnosis due to their strong capability in extracting local features. Ince et al. [5] proposed a one-dimensional CNN for real-time motor fault detection, demonstrating the effectiveness of end-to-end learning. In addition, Chen et al. [6] utilized CNN-based models for gearbox fault identification, further verifying the applicability of deep architectures in mechanical systems.

To improve feature extraction capability, several enhanced CNN architectures have been proposed. Zhang et al. [7] introduced the wide first-layer convolutional neural network (WDCNN), which improves robustness by enlarging the receptive field. He et al. [8] proposed residual learning, which facilitates the training of deeper networks and has been widely adopted in fault diagnosis tasks. Furthermore, Lei et al. [9] reviewed recent advances in machine learning-based fault diagnosis and highlighted the importance of robust feature representation under complex industrial environments.

Despite these advances, the presence of complex noise in industrial environments still poses significant challenges. Noise interference may distort fault-related patterns and degrade diagnostic performance. To address this issue, Zhao et al. [10] proposed the deep residual shrinkage network (DRSN), which introduces adaptive thresholding to suppress noise-related features. In addition, Li et al. [11] developed an ensemble CNN model with improved evidence fusion, while Verstraete et al. [12] employed time–frequency analysis combined with deep learning to enhance robustness under noisy conditions.

Another critical challenge arises from the mismatch between training and testing conditions. In real-world applications, the noise distribution of testing data is often different from that of training data, leading to severe performance degradation. To alleviate this issue, domain adaptation and transfer learning methods have been investigated. Ganin and Lempitsky [13] proposed domain-adversarial training to learn domain-invariant features, while Pan and Yang [14] provided a comprehensive survey on transfer learning. However, these methods mainly focus on global distribution alignment and may not fully address sample-level variability under heterogeneous degradation conditions.

Recently, several representative studies have further explored fault diagnosis under limited-label, cross-domain, and heterogeneous-condition scenarios. Wang et al. proposed a self-supervised time–frequency dual-domain prediction framework (TFDDP) for train bearing fault diagnosis, which improves representation learning capability through dual-domain self-supervised prediction tasks [15]. Furthermore, a time–frequency dual-domain contrast and fusion framework (TFDDCF) was introduced for vehicle bearing fault diagnosis, where contrastive learning and dual-domain fusion strategies were jointly employed to improve feature consistency and discriminative capability under limited-label conditions [16]. In addition, digital twin-assisted graph contrastive domain adaptation (GCDA) has been investigated for small-sample bearing fault diagnosis, where graph-based domain alignment and digital twin information are utilized to improve cross-domain generalization performance [17]. These studies demonstrate the growing interest in self-supervised learning, adaptive representation learning, and domain generalization for intelligent fault diagnosis under complex industrial environments.

However, most existing methods mainly focus on global representation enhancement, domain alignment, or self-supervised pretraining, while the adaptive balancing of complementary feature representations under sample-wise heterogeneous degradation conditions remains insufficiently explored. In practical scenarios, the degree of noise contamination may vary significantly across different samples. Some samples may retain relatively clear fault characteristics, while others are heavily corrupted by noise. Under such conditions, a unified feature extraction strategy may become insufficient. Models optimized for clean data may fail under severe degradation conditions, whereas aggressive noise suppression may also remove useful fault-related structural information. This limitation indicates the necessity of developing adaptive mechanisms that can dynamically adjust feature utilization according to the reliability characteristics of different input samples.

Motivated by the above observations, this paper proposes a reliability-guided adaptive feature fusion framework (RGAF-Net) for bearing fault diagnosis under cross-noise conditions. The proposed method introduces a dual-path feature learning architecture to model complementary representation tendencies, including globally stable semantic representations and detail-sensitive local structural responses. Furthermore, a lightweight reliability estimation branch and a sample-wise adaptive routing mechanism are employed to dynamically balance dual-path feature contributions under different degradation conditions. Unlike existing methods that mainly rely on fixed feature extraction or global domain alignment, the proposed framework focuses on adaptive feature balancing under heterogeneous sample-level degradation conditions.

The main contributions of this paper are summarized as follows:

1. A reliability-guided adaptive feature fusion framework is proposed to improve fault diagnosis robustness under heterogeneous cross-noise conditions;
2. A dual-path feature learning architecture with different aggregation tendencies is designed to jointly exploit globally robust semantic representations and complementary local structural responses;
3. A lightweight reliability-aware routing mechanism is introduced to perform sample-wise adaptive feature balancing according to degradation-sensitive reliability descriptors;
4. Extensive experiments on the PU and JNU datasets demonstrate that the proposed method achieves improved robustness and feature discriminability under mismatched noise environments.

The remainder of this paper is organized as follows. Section 2 reviews related work. Section 3 introduces the proposed method. Section 4 presents experimental results and analysis. Section 5 concludes the paper.

## 2. Related Work

This section reviews representative studies related to this work from three aspects: CNN-based fault diagnosis methods, noise-robust learning strategies, and domain generalization techniques. Unlike the Introduction, which focuses on problem motivation, this section emphasizes methodological development and their limitations.

### 2.1. CNN-Based Fault Diagnosis Methods

With the advancement of deep learning, convolutional neural networks (CNNs) have been widely adopted in fault diagnosis tasks due to their strong capability in automatic feature extraction. Wen et al. [18] proposed a data-driven fault diagnosis method based on CNN, demonstrating that deep architectures can effectively learn discriminative features directly from raw signals. Guo et al. [19] further explored recurrent neural networks for bearing health indicator construction, highlighting the potential of deep models in capturing temporal dependencies.

In addition to standard CNN architectures, various improved models have been developed to enhance diagnostic performance. Wang et al. [20] reviewed vibration-based health indicators and emphasized the importance of effective feature representation in prognostics and health management. Wang et al. [21] proposed a two-stage data-driven framework for bearing degradation analysis, combining feature extraction and prediction in a unified model.

More recently, advanced architectures incorporating attention mechanisms and multi-scale learning have been investigated. For example, Wang et al. [22] introduced a multi-head attention-based CNN to enhance feature representation by capturing global dependencies. These studies demonstrate that architectural improvements can significantly improve model performance.

However, most existing CNN-based methods adopt a single-path feature extraction paradigm, where all samples are processed using a fixed representation strategy. Such designs may be insufficient when dealing with heterogeneous noise conditions, as they lack flexibility in adapting to varying input quality.

### 2.2. Noise-Robust Fault Diagnosis Methods

In practical industrial environments, vibration signals are often contaminated by complex noise, which significantly degrades diagnostic performance. To address this issue, various noise-robust learning strategies have been proposed.

Traditional approaches combine signal processing techniques with machine learning models. For instance, Zhang et al. [23] integrated permutation entropy, ensemble empirical mode decomposition, and support vector machines to enhance feature robustness under noisy conditions. Although effective, such methods rely heavily on handcrafted features and prior knowledge.

With the development of deep learning, noise-robust models have been further explored. Wang and Cao [24] proposed a multiscale convolutional neural network to improve feature extraction under complex noise environments. Similarly, Liu et al. [25] introduced a multi-domain feature learning framework based on CNN, which captures complementary information from different representations.

In addition, feature fusion strategies have been widely studied to improve robustness. Dong and Lotfipoor [26] proposed a feature fusion-based method that combines dilated CNN and multi-domain signal processing to enhance diagnostic accuracy. Qiu et al. [27] provided a comprehensive review of deep learning techniques in fault diagnosis and highlighted the importance of multi-feature integration.

Despite these advances, most noise-robust methods focus on improving global feature representation and implicitly assume that training and testing noise distributions are similar. As a result, their performance may degrade under cross-noise conditions, where the noise characteristics differ significantly between domains.

### 2.3. Domain Generalization for Fault Diagnosis

To address distribution mismatch between training and testing data, domain adaptation and domain generalization methods have been introduced into fault diagnosis.

Long et al. [28] proposed deep adaptation networks to learn transferable features across domains by minimizing distribution discrepancies. Tzeng et al. [29] further developed adversarial discriminative domain adaptation, which aligns feature distributions through adversarial training. These methods have laid the foundation for domain-invariant feature learning.

In the context of fault diagnosis, domain adaptation techniques have been applied to improve model robustness. Shao and Kim [30] proposed an unsupervised domain adaptive 1D-CNN for bearing fault diagnosis, demonstrating the effectiveness of domain adaptation under varying conditions. Liu et al. [31] introduced a multi-adversarial domain adaptation framework to enhance generalization performance across different operating environments.

Although these methods reduce distribution discrepancies at the domain level, they mainly focus on global feature alignment and assume that samples within a domain share similar characteristics. However, in cross-noise scenarios, the degree of noise contamination may vary significantly across individual samples. Enforcing a unified domain-invariant representation may therefore lead to suboptimal performance.

### 2.4. Summary and Limitations

In summary, existing studies have made significant progress in fault diagnosis through improved CNN architectures, noise-robust feature learning, and domain generalization techniques. However, two key limitations remain:

1. Most methods rely on fixed feature extraction or fusion strategies, which lack flexibility in handling sample-wise variations under heterogeneous noise conditions;
2. Existing approaches rarely incorporate explicit mechanisms to evaluate feature reliability and adapt feature utilization accordingly.

These limitations motivate the development of a sample-wise adaptive feature fusion framework, which forms the basis of the proposed method.

## 3. Proposed Method

### 3.1. Overall Framework

In cross-noise fault diagnosis scenarios, the noise distribution of training data is often inconsistent with that of testing data, leading to severe feature degradation and reduced model generalization ability. Given multiple source domains with different noise conditions, denoted as $D_{s}=\left\{D_{1},D_{2},\ldots ,D_{k}\right\}$, the objective is to learn a mapping function f:x→y that can generalize to an unseen target domain $D_{t}$, where the target noise condition is unavailable during training.

Under heterogeneous noise environments, different samples may exhibit different degradation tendencies, resulting in inconsistent feature reliability across samples. Consequently, fixed feature extraction and static fusion strategies may become less effective when facing mismatched noise conditions.

**Figure 1:**
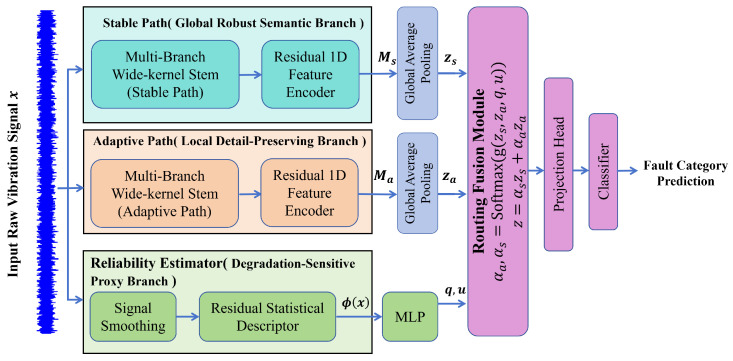
Overall architecture of the proposed RGAF-Net framework. The model consists of dual-path WDCNN-based feature extractors, a reliability estimation branch, and a sample-wise adaptive routing fusion mechanism.

To address this issue, the proposed Reliability-Guided Adaptive Feature Fusion Network (RGAF-Net) introduces a dual-path feature learning framework together with a reliability-aware adaptive routing mechanism, as illustrated in Figure 1.

Given an input vibration signal $x\in R^{L}$, the framework first feeds the signal into two parallel WDCNN-based feature extraction pathways with different architectural biases. The stable path mainly focuses on extracting globally consistent structural representations through deeper hierarchical aggregation and early feature compression, while the adaptive path aims to preserve local detail-sensitive structural responses through delayed pooling and enhanced local feature interactions.

The extracted feature maps from the two pathways are denoted as

(1) $$M_{s}=F_{s}\left(x\right),\;M_{a}=F_{a}\left(x\right)$$

where $F_{s}\left(\cdot \right)$ and $F_{a}\left(\cdot \right)$ represent the stable-path and adaptive-path feature extractors, respectively.

After global average pooling, the final pathway representations are obtained as

(2) $$z_{s}=GAP\left(M_{s}\right),\;z_{a}=GAP\left(M_{a}\right)$$

Meanwhile, a lightweight reliability estimation branch extracts statistical descriptors from the input signal to characterize degradation-sensitive reliability tendencies. The extracted reliability descriptors are further used to dynamically guide feature fusion under different noise conditions. Therefore, the proposed framework can be viewed as a three-branch collaborative architecture, consisting of a stable global representation pathway, an adaptive structural pathway, and a reliability estimation pathway for degradation-aware adaptive fusion.

Based on the dual-path features and reliability descriptors, a sample-wise routing module generates adaptive routing weights for dynamic feature balancing. Unlike channel-wise attention mechanisms, the proposed routing strategy performs sample-level adaptive fusion between the two feature pathways.

Finally, the fused feature representation is fed into the classification head for fault category prediction:

(3) $${\hat{y}}_{i}=Softmax\left(G\left(f_{i}\right)\right)$$

where G(·) denotes the fully connected classification head, and ${\hat{y}}_{i}$ represents the predicted class probability distribution of the *i*-th sample.

It should be noted that the proposed reliability estimation module serves as a lightweight degradation-aware reliability proxy for guiding adaptive feature fusion under cross-noise conditions.For clarity, the major symbols and notations used throughout the proposed method are summarized in Table 1.

### 3.2. Dual-Path WDCNN-Based Feature Learning

To improve representation robustness under heterogeneous noise conditions, RGAF-Net adopts a dual-path WDCNN-based feature learning architecture. The two pathways share a similar wide-convolution backbone structure, but introduce different feature aggregation and pooling strategies, thereby producing complementary representation tendencies under noisy environments.

Both pathways employ multi-branch wide convolutional frontends with kernel sizes of 64, 32, and 16 to capture multi-scale temporal patterns from the raw vibration signal. The wide convolution design enlarges the effective receptive field, allowing the network to capture long-range periodic fault-related patterns while reducing sensitivity to local random perturbations.

After multi-branch feature extraction, the two pathways adopt different structural organizations for feature propagation and aggregation, leading to different representation preferences under degraded conditions.

#### 3.2.1. Stable Path

**Figure 2:**
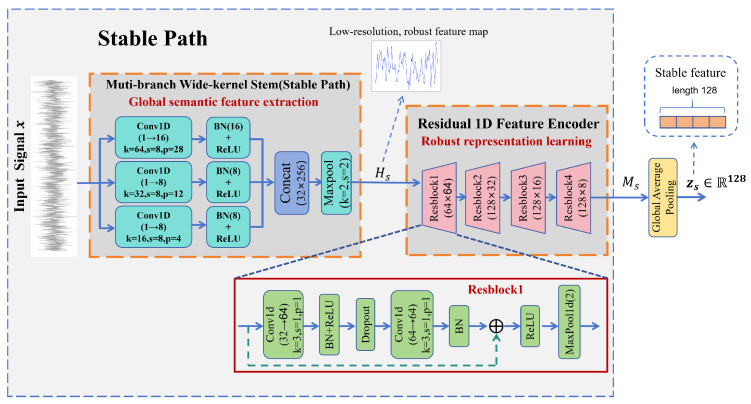
Structure of the stable path. The pathway mainly focuses on extracting globally consistent robust representations through early feature compression and deep hierarchical aggregation.

The structure of the stable path is illustrated in Figure 2.

In the stable path, multi-branch convolutional features are first concatenated and then compressed through early max-pooling operations before entering multiple residual blocks.

After the first pooling stage, an intermediate feature representation is obtained and denoted as

(4) $$H_{s}=F_{s}^{\left(1\right)}\left(x\right)$$

where $H_{s}\in R^{C_{1}\times T_{1}}$ denotes the intermediate pooled feature map of the stable path.

The stable-path feature extraction process can be formulated as

(5) $$M_{s}=F_{s}\left(x\right)$$

where $M_{s}\in R^{C\times T}$ denotes the intermediate feature map extracted by the stable pathway.

Compared with shallow local feature interactions, the stable path employs deeper hierarchical residual aggregation after feature compression, which encourages the network to focus more on globally aggregated and relatively stable structural patterns. The early pooling operation further suppresses local high-frequency perturbations caused by severe noise contamination. Since random noise usually exhibits stronger high-frequency local fluctuations than fault-related periodic structures, early feature compression helps improve global representation stability.

Therefore, the stable pathway mainly tends to preserve globally robust fault-related representations and improve representation stability under strong noise conditions.

#### 3.2.2. Adaptive Path

**Figure 3:**
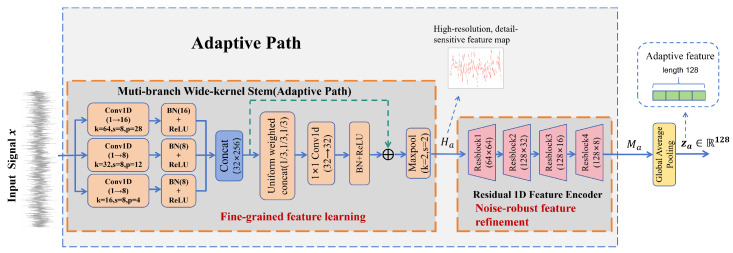
Structure of the adaptive path. The pathway mainly focuses on preserving complementary detail-sensitive structural responses through delayed pooling and enhanced local feature interactions.

The structure of the adaptive path is illustrated in Figure 3.

Unlike the stable path, the adaptive path introduces additional local feature interaction operations before feature compression. After multi-branch convolutional extraction, the feature maps are first processed through residual interaction modules and uniform weighted feature concatenation before the subsequent pooling operation.

After delayed pooling and local interaction operations, the adaptive pathway generates an intermediate feature representation:

(6) $$H_{a}=F_{a}^{\left(1\right)}\left(x\right)$$

where $H_{a}\in R^{C_{1}\times T_{1}}$ denotes the intermediate pooled feature map of the adaptive path.

Compared with $H_{s}$, the intermediate response $H_{a}$ preserves richer local structural activation patterns before deep hierarchical aggregation.

Specifically, feature maps from different convolution branches are aggregated with equal weighting coefficients to preserve balanced multi-scale local responses before feature compression. This operation avoids excessive dominance of a single receptive-field scale and helps retain diverse local structural characteristics in the latent feature space.

The adaptive-path feature extraction process is formulated as

(7) $$M_{a}=F_{a}\left(x\right)$$

where $M_{a}\in R^{C\times T}$ denotes the adaptive-path feature representation.

Specifically, the delayed pooling strategy preserves more local temporal structures before feature compression, while the intermediate feature interaction operations enhance local structural response diversity in the latent feature space.

Compared with the stable path, the adaptive path maintains relatively stronger sensitivity to transient local variations and fine-grained structural cues before hierarchical aggregation. Such detail-sensitive responses may provide complementary information beyond globally aggregated robust representations.

Under moderate degradation conditions, local fault-related transient structures may still remain partially distinguishable. In such cases, preserving additional local structural responses can provide useful complementary cues for fault discrimination.

However, under extremely severe noise contamination, excessive sensitivity to local responses may also introduce unstable noise-sensitive perturbations. Consequently, the adaptive path is not intended to serve as the primary noise-robust representation extractor. Instead, it mainly acts as a complementary detail-preserving branch that provides additional local structural information beyond globally stable structural representations.

It should be noted that the adaptive path does not perform explicit signal denoising, signal restoration, or frequency-domain filtering. Instead, it improves representation flexibility by preserving complementary local structural responses through delayed aggregation and local feature interaction mechanisms.

By combining the complementary representations generated by the two pathways, the proposed framework jointly exploits globally robust structural information and detail-sensitive local structural responses, thereby improving feature representation flexibility under heterogeneous cross-noise conditions.

Importantly, the proposed dual-path architecture does not aim to explicitly disentangle semantic components or perform signal restoration. Instead, the two pathways provide different representation biases through distinct feature aggregation and pooling strategies, allowing the framework to dynamically balance global robustness and local detail preservation under different degradation conditions. Structural differences and representation tendencies of the two feature pathways are summarized in Table 2.

#### 3.2.3. Reliability Estimation

To characterize degradation-sensitive signal tendencies without introducing significant computational overhead, a lightweight statistical reliability estimation branch is employed.

Given an input vibration signal *x*, a statistical descriptor vector ϕ(x) is first extracted from the raw signal:

(8) $$\phi \left(x\right)\in R^{m}$$

where *m* denotes the number of handcrafted statistical descriptors.

The descriptor vector includes both time-domain and frequency-domain statistical characteristics, such as root mean square (RMS), kurtosis, skewness, peak-to-peak value, spectral energy distribution, and frequency-domain variance, which reflect signal fluctuation intensity and degradation-sensitive variations under different noise conditions.

The extracted statistical descriptor is further projected through a lightweight multi-layer perceptron:

(9) $$q_{i}=H_{1}\left(\phi \left(x_{i}\right)\right)$$

where $q_{i}\in R^{d}$ denotes the reliability descriptor vector of the *i*-th sample. During mini-batch training, all reliability descriptor vectors are stacked into the batch-wise matrix representation:

(10) $$Q={\left[q_{1},q_{2},\ldots ,q_{B}\right]}^{T}\in R^{B\times d}$$

Subsequently, a compact scalar reliability indicator is obtained as

(11) $$u_{i}=H_{2}\left(q_{i}\right)$$

where $u_{i}\in \left[0,1\right]$.

Here, $H_{1}\left(\cdot \right)$ and $H_{2}\left(\cdot \right)$ are lightweight fully connected projection networks, and the scalar output $u_{i}$ is normalized through a sigmoid activation function.

The descriptor vector $q_{i}$ captures multi-dimensional degradation-sensitive characteristics, while the scalar indicator $u_{i}$ provides a compact reliability-aware estimation of the input sample. Larger $u_{i}$ values indicate stronger estimated degradation tendency and lower feature reliability.

**Figure 4:**
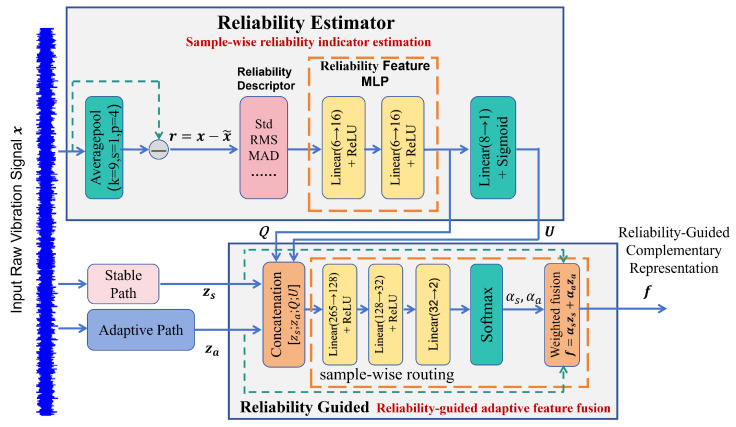
Structure of the proposed reliability-guided dynamic routing mechanism. The routing module generates sample-wise adaptive fusion coefficients based on dual-path feature representations and reliability descriptors, enabling dynamic soft balancing between globally stable structural features and detail-sensitive local structural features under different noise conditions.

During mini-batch training with batch size *B*, all scalar reliability indicators are stacked into the batch-wise vector representation:

(12) $$U={\left[u_{1},u_{2},\ldots ,u_{B}\right]}^{T}\in R^{B\times 1}$$

which corresponds to the vector representation illustrated in Figure 4.

It should be noted that the proposed reliability estimation module does not aim to model uncertainty in a strict Bayesian or probabilistic sense. Instead, the estimated descriptors serve as lightweight degradation-aware reliability proxies for guiding adaptive feature fusion under cross-noise conditions.

#### 3.2.4. Dynamic Routing and Feature Fusion

The overall structure of the proposed reliability-guided routing mechanism is illustrated in Figure 4.

Based on the extracted pathway representations $z_{s,i}$, $z_{a,i}$, together with the reliability descriptors $q_{i}$ and $u_{i}$, the routing network generates two normalized routing coefficients for the *i*-th sample:

(13) $$\left[\alpha_{s,i},\alpha_{a,i}\right]=g\left(\left[z_{s,i},z_{a,i},q_{i},u_{i}\right]\right)$$

where $\alpha_{s,i},\alpha_{a,i}\in \left[0,1\right]$ and $\alpha_{s,i}+\alpha_{a,i}=1$. and g(·) denotes a lightweight fully connected routing network.

The routing coefficient $\alpha_{i}$ is a sample-wise scalar weight used to dynamically balance the contributions of the stable pathway and adaptive pathway during feature fusion.

For a mini-batch with size *B*, the routing coefficients are represented as

(14) $$\alpha ={\left[\alpha_{1},\alpha_{2},\ldots ,\alpha_{B}\right]}^{T}\in R^{B\times 1}$$

During feature fusion, the scalar routing coefficient of each sample is automatically broadcast along the feature dimension to match the size of pathway feature vectors. Therefore, the routing operation is performed in a sample-wise manner rather than channel-wise or element-wise weighting.

The fused representation of the *i*-th sample is formulated as

(15) $$f_{i}=\alpha_{s,i}z_{s,i}+\alpha_{a,i}z_{a,i}$$

where $f_{i}\in R^{C}$.

Under severely degraded conditions, the routing mechanism tends to assign larger weights to the stable pathway, since globally aggregated structural representations are generally more resistant to strong noise contamination.

In contrast, under relatively clean or mildly degraded conditions, the adaptive pathway can provide additional local structural details and complementary fine-grained responses for feature enhancement.

It mainly serves as an adaptive feature balancing strategy for improving fusion flexibility under heterogeneous noise conditions. The primary performance improvement still originates from the enhanced dual-path feature extraction architecture.

It should be emphasized that the proposed routing mechanism performs dynamic soft balancing rather than hard pathway selection. Even under severe noise contamination, the adaptive pathway still preserves partial contributions, since residual local structural responses may still contain complementary discriminative information beyond globally aggregated structural representations.

This sample-dependent routing behavior is further validated in the experimental visualization analysis under different noise conditions.

### 3.3. Optimization Objective

The proposed RGAF-Net is trained in an end-to-end manner using a joint optimization objective composed of a classification loss and two routing-related regularization terms. The overall objective is designed to simultaneously ensure fault classification performance and stable adaptive fusion behavior. For the *i*-th sample, let $y_{i}\in R^{N_{c}}$ denote the one-hot ground-truth label vector and ${\hat{y}}_{i}\in R^{N_{c}}$ denote the predicted class probability vector generated by the classifier. Let $w_{i}$ represent the class-balancing weight corresponding to the category of the current sample. The routing coefficients generated by the routing module are denoted as $\alpha_{s,i}$ and $\alpha_{a,i}$, which satisfy $\alpha_{s,i},\alpha_{a,i}\in \left[0,1\right]$ and $\alpha_{s,i}+\alpha_{a,i}=1$.

The weighted classification loss is formulated as

(16) $$L_{cls}=-\frac{1}{B}\sum_{i=1}^{B}w_{i}\sum_{c=1}^{N_{c}}y_{i,c}\log\left({\hat{y}}_{i,c}\right)$$

where *B* denotes the mini-batch size and $N_{c}$ denotes the number of fault categories.

Since the proposed framework employs adaptive dual-path feature fusion, the routing module may excessively rely on a single feature pathway during training, leading to routing collapse and reduced representation diversity. To alleviate this issue, a routing balance regularization term is introduced:

(17) $$L_{bal}={\left({\bar{\alpha }}_{s}-\rho \right)}^{2}$$

where

(18) $${\bar{\alpha }}_{s}=\frac{1}{B}\sum_{i=1}^{B}\alpha_{s,i}$$

denotes the average routing weight assigned to the stable pathway within the current mini-batch, and ρ∈(0,1) is a predefined routing balance factor.

Since $\alpha_{a,i}=1-\alpha_{s,i}$, only the stable-path routing coefficient is explicitly regularized for simplicity.

In addition, an entropy regularization term is employed to encourage non-trivial routing behavior and improve routing diversity across different samples:

(19) $$L_{ent}=-\frac{1}{B}\sum_{i=1}^{B}\left[\alpha_{s,i}\log\left(\alpha_{s,i}\right)+\alpha_{a,i}\log\left(\alpha_{a,i}\right)\right]$$

The entropy term prevents the routing coefficients from collapsing to deterministic extreme values over all samples, thereby improving the flexibility of adaptive feature fusion under heterogeneous noise conditions.

Finally, the overall optimization objective of RGAF-Net is formulated as

(20) $$L=L_{cls}+\lambda_{1}L_{bal}+\lambda_{2}L_{ent}$$

where $\lambda_{1}$ and $\lambda_{2}$ are trade-off hyperparameters used to balance the contributions of different optimization terms.

It should be emphasized that the routing-related regularization terms are not designed to directly improve feature extraction capability. Instead, they are introduced to stabilize the adaptive fusion behavior and prevent the routing module from degenerating into fixed-path feature selection during training.

## 4. Experiments and Analysis

In this section, extensive experiments are conducted to evaluate the effectiveness of the proposed reliability-aware dual-path framework under cross-noise conditions. The experiments are performed on two widely used bearing datasets, i.e., the PU dataset and the JNU dataset. The evaluation focuses on diagnostic performance, robustness under varying noise levels, and the effectiveness of the proposed reliability-aware routing mechanism.

### 4.1. Experimental Setup

#### 4.1.1. Datasets

To comprehensively evaluate the diagnostic performance of the proposed method under cross-noise conditions, two publicly available bearing fault datasets, i.e., the PU dataset and the JNU dataset, are adopted in this study.

The PU dataset is collected by Paderborn University [32], which includes healthy bearings, artificially damaged bearings, and real degradation bearings. Among them, the real damaged bearings are obtained through accelerated lifetime tests and exhibit more complex degradation characteristics, making this dataset more challenging for fault diagnosis tasks [33]. The sampling frequency of the PU dataset is 64 kHz.

In this study, only the real damaged bearings are selected to better reflect practical industrial scenarios. To eliminate the influence of varying operating conditions, a fixed working condition (N09M07F10) is adopted. Based on the selected data, a multi-class classification task consisting of 14 categories (including healthy and various fault types) is constructed. The detailed category definitions are listed in Table 3.

The selected categories cover diverse fault types and damage mechanisms, which increases the difficulty of the classification task and better reflects real-world industrial scenarios.This experimental setting introduces significant challenges due to noise distribution mismatch, making it suitable for evaluating model generalization ability.

The JNU dataset is provided by Jiangnan University [34]. It contains vibration signals collected from bearings with artificially introduced faults using electrical discharge machining, including inner race faults, outer race faults, and rolling element faults. The experiments are conducted under three rotational speeds (600 rpm, 800 rpm, and 1000 rpm), with a sampling frequency of 50 kHz.This dataset is one of the more difficult datasets in the field of bearing fault diagnosis [33].

To accommodate variable-speed conditions, the dataset is organized into 10 categories, including different fault types under multiple rotational speeds as well as normal conditions. Due to the relatively small dataset size, an overlapping sliding window strategy is adopted with a step size of 100 to increase data utilization.

#### 4.1.2. Data Preprocessing and Sample Construction

All raw vibration signals are segmented into samples with a fixed length of 2048. For the PU dataset, non-overlapping segmentation is applied due to its well-structured data organization. For the JNU dataset, a sliding window strategy is employed to generate overlapping samples. Each sample is represented as a one-dimensional vector of length 2048 and fed into the network as a single-channel input.

After sample construction, all data are grouped by category and split into training, validation, and testing sets with a ratio of 7:2:1. The validation set is used for model selection and early stopping, while the test set is only used for final performance evaluation.

#### 4.1.3. Noise Injection and Cross-Noise Protocol

To simulate realistic industrial environments where training and testing noise distributions differ, a cross-noise diagnosis setting is constructed.Additive Gaussian white noise is introduced to the vibration signals to generate noisy samples. The noise level is controlled by the signal-to-noise ratio (SNR).

For the PU dataset, the training set (source domain) includes noise levels of 2 dB, 0 dB, and −2 dB, while the testing set (target domain) is set to a more severe noise level of −4 dB. For the JNU dataset, the training noise levels are set to −4 dB, −6 dB, and −8 dB, while the testing noise level is set to −10 dB.

This setting ensures that the model does not observe the target noise distribution during training, thus providing a fair evaluation of cross-noise generalization ability.

#### 4.1.4. Implementation Details

The proposed model is implemented using PyTorch 2.4.1 and optimized with the Adam optimizer [35]. The initial learning rate is set to $1\times 10^{-3}$, and a combination of warm-up and cosine decay scheduling is applied during training.

The batch size is set to 64. To improve robustness against noise variations, lightweight time-domain perturbation and Mixup data augmentation [36] (with α=0.2) are adopted during training. In the testing phase, test-time augmentation (TTA) is used for prediction fusion.

To reduce randomness, all experiments are repeated three times under fixed random seeds, and the average results are reported.

#### 4.1.5. Evaluation Metrics

To comprehensively evaluate model performance, three metrics are adopted, including Accuracy, Macro-F1, and Macro-FPR.Macro-averaging is adopted to mitigate the influence of potential class imbalance.

Accuracy measures the overall classification performance, while Macro-F1 reflects the balanced performance across different categories. Macro-FPR evaluates the false alarm rate under noisy conditions, which is particularly important in practical fault diagnosis applications.

In addition, confusion matrices and t-SNE visualization are used to analyze the feature distribution in the target domain.

#### 4.1.6. Comparison Methods

To validate the effectiveness of the proposed method, several representative fault diagnosis models are selected for comparison, including WDCNN [7], DRSN-1D [10], MSCNN-1D [37], ResNet-1D-Small [8], and DARM [38].

These methods represent different technical paradigms such as wide-kernel convolution, residual shrinkage, multi-scale feature extraction, and domain generalization. All comparison models are implemented under the same data partition, cross-noise settings, and training strategies to ensure fairness and comparability of experimental results.

### 4.2. Performance Trend and Mechanism Analysis Under Different Noise Levels

#### 4.2.1. Performance Degradation Trend

**Figure 5:**
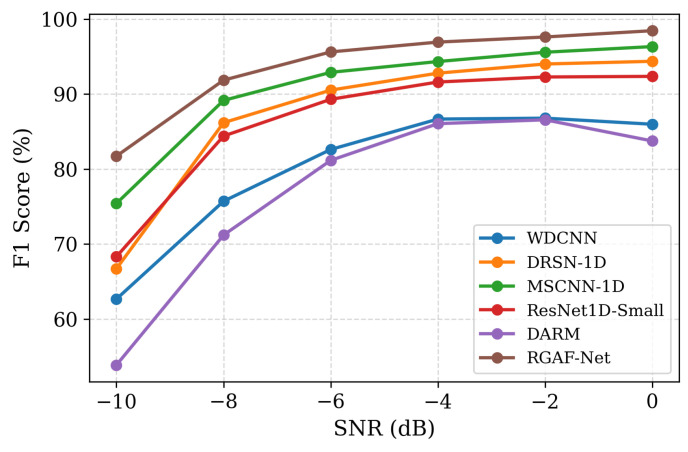
Performance comparison of different methods under varying SNR levels.

To analyze the robustness of the proposed method under varying noise conditions, the performance trends of different models across multiple SNR levels are illustrated in Figure 5.

As the SNR decreases, the performance of all comparison methods exhibits a clear degradation trend, which is consistent with the increased difficulty of feature extraction under stronger noise contamination [39]. However, compared with baseline methods, the proposed RGAF-Net shows a significantly slower performance degradation rate.

In particular, in the low-SNR region (from −6 dB to −10 dB), most baseline models suffer substantial performance drops, while RGAF-Net maintains relatively high accuracy, demonstrating superior robustness under extreme noise conditions.

#### 4.2.2. Pathway Feature Response Analysis

**Figure 6:**
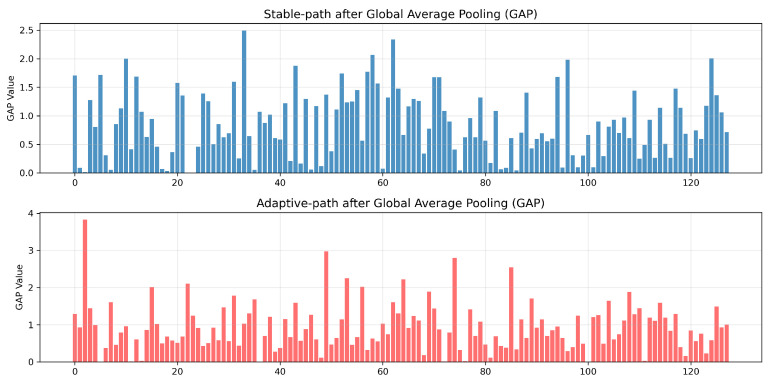
Comparison of GAP feature activation responses of the stable pathway and adaptive pathway under severe noise conditions.

To further investigate the representation characteristics of the two feature extraction pathways, the GAP feature activation responses under severe noise conditions are visualized in Figure 6. The visualization is generated from representative test samples under the most challenging target-domain noise condition (SNR = −10 dB).

Figure 6 shows that the two pathways exhibit noticeably different activation distributions in the latent feature space. The stable pathway produces relatively sparse activation patterns, where stronger responses are concentrated in a limited number of channels while many channels remain weakly activated. This behavior is consistent with the representation tendency of the stable pathway, which emphasizes globally aggregated and relatively noise-insensitive semantic responses after early feature compression and hierarchical aggregation.

In contrast, the adaptive pathway exhibits denser activations across more feature dimensions, with multiple channels showing relatively strong responses simultaneously. Such activation patterns suggest that the delayed pooling strategy and local interaction operations preserve more diverse local structural responses in the latent feature space.

The distinct activation distributions indicate that the two pathways introduce different representation biases under noisy conditions. The stable pathway tends to generate more compact and globally stable responses, whereas the adaptive pathway maintains richer detail-sensitive structural activations.

These complementary characteristics help explain the effectiveness of the proposed dual-path architecture. By jointly exploiting globally robust structural representations and locally detail-sensitive structural responses, the proposed framework achieves improved representation flexibility under heterogeneous cross-noise conditions.

#### 4.2.3. Reliability Estimation Behavior

**Figure 7:**
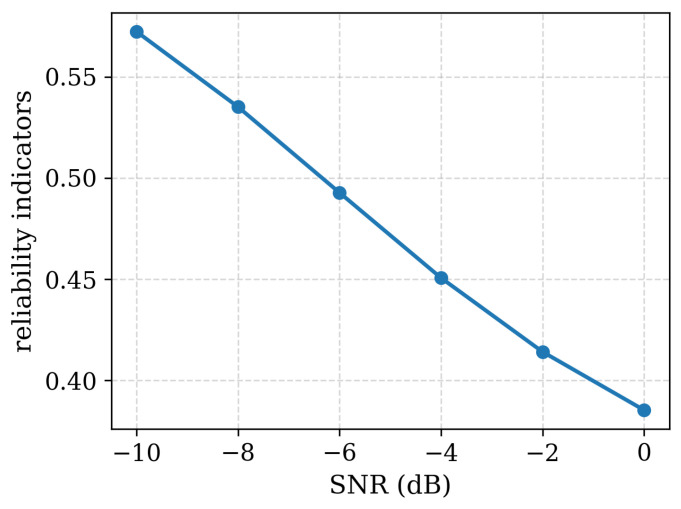
Variation of reliability indicators under different SNR levels.

To further investigate the internal mechanism, the variation of reliability estimation under different noise levels is illustrated in Figure 7.

It can be observed that the reliability indicator increases as the SNR decreases, indicating that the proposed reliability estimator effectively captures the degree of noise contamination in vibration signals.

#### 4.2.4. Dynamic Routing and Path Weight Analysis

**Figure 8:**
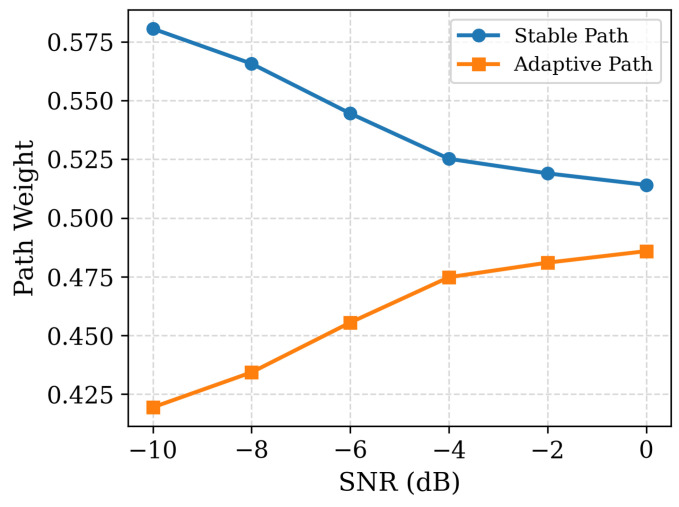
Average routing weights of the stable pathway and adaptive pathway under different noise levels.

The variation of dynamic routing weights under different noise levels is shown in Figure 8. The results show that the routing weights change smoothly across different noise conditions. Interestingly, under extremely severe noise conditions, the routing mechanism tends to assign relatively larger weights to the stable pathway.This indicates that globally stable structural representations become more reliable than highly detail-sensitive local responses when the signal is heavily corrupted.

#### 4.2.5. Mechanism Interpretation

This behavior can be explained from the perspective of feature reliability. The adaptive path can preserve more local detail-sensitive structural responses under moderate degradation conditions. However, under extremely severe noise, it may amplify noise components, making the stable path more reliable for classification.Therefore, the model adaptively shifts its reliance toward the stable path, reflecting a reliability-aware routing mechanism rather than a simple noise-driven adjustment.

This behavior is also consistent with the architectural characteristics of the two pathways. The stable pathway performs earlier feature compression and deeper hierarchical aggregation, which suppresses local perturbations and preserves globally stable structural patterns. In contrast, the adaptive pathway maintains stronger sensitivity to local structural variations, which may become less reliable when the signal is heavily contaminated by extreme noise.

#### 4.2.6. Overall Discussion on Performance Trend and Mechanism Analysis

Overall, the proposed framework establishes a progressive adaptive process:

Input Degradation Tendency → Reliability Estimation → Adaptive Routing → Feature Fusion Adjustment

As the input degradation condition changes, the estimated reliability descriptors dynamically influence the routing behavior between the two feature pathways. This enables the framework to adaptively balance globally stable structural representations and detail-sensitive local structural responses under heterogeneous noise conditions.

The visualization results further support the effectiveness of the proposed reliability-guided adaptive fusion mechanism.

### 4.3. Comparison with State-of-the-Art Methods

To comprehensively evaluate the effectiveness of the proposed method, comparisons with several representative models are conducted on both the PU and JNU datasets under cross-noise conditions.

#### 4.3.1. Results on the PU Dataset

The comparison results on the PU dataset are presented in Table 4.

It can be observed that the proposed RGAF-Net achieves the best performance across all evaluation metrics. Specifically, it obtains an Accuracy of 95.71% and a Macro-F1 score of 93.15%, outperforming the classical WDCNN by 6.44 and 4.14 percentage points, respectively. Meanwhile, the Macro-FPR is significantly reduced from 0.82 to 0.45, indicating a substantial decrease in false alarms.

Compared with more advanced models such as DRSN-1D and MSCNN-1D, the proposed method still maintains clear advantages, demonstrating that the improvement is not limited to a specific baseline. In addition, although DARM is designed for domain generalization, RGAF-Net still achieves better performance across all metrics, suggesting that the proposed reliability-guided feature routing mechanism is more effective in handling cross-noise distribution shifts.

Overall, the results on the PU dataset demonstrate that the proposed method can simultaneously improve classification accuracy and reduce misclassification risk, which is critical for practical fault diagnosis applications.

#### 4.3.2. Results on the JNU Dataset

The comparison results on the JNU dataset are shown in Table 5.

Compared with the PU dataset, the JNU task involves more severe target noise conditions (−10 dB), making it significantly more challenging.

Despite the increased difficulty, RGAF-Net still achieves the best performance, with an Accuracy of 84.06% and a Macro-F1 score of 81.73%. Compared with WDCNN, the proposed method improves Accuracy by 18.94 percentage points and Macro-F1 by 19.05 percentage points, while significantly reducing the Macro-FPR.

It is also observed that the performance gap between different methods becomes more pronounced under such extreme noise conditions. Most baseline models suffer substantial performance degradation, whereas the proposed method maintains relatively stable performance.

It is worth noting that the performance of DARM degrades significantly on this dataset. A possible explanation is that DARM mainly focuses on learning globally shared representations across domains.Under extremely severe noise contamination, the signal structure becomes highly distorted, which may reduce the effectiveness of global feature alignment strategies.

In contrast, the proposed method adopts a sample-wise adaptive routing mechanism guided by reliability estimation, enabling more flexible and fine-grained feature fusion. This allows the model to better handle highly corrupted and sample-dependent signals, leading to superior performance under extreme noise conditions.

#### 4.3.3. Overall Discussion

Based on the results of the two datasets, it can be concluded that the proposed RGAF-Net consistently outperforms the comparison methods even in the presence of cross-noise.

The performance improvement can be attributed to three main factors. First, the multi-branch wide-kernel front-end enhances multi-scale feature extraction, leading to more robust representations. Second, the dual-path architecture enables complementary modeling of stable semantic features and local detail-sensitive structural features. Third, the reliability-guided routing mechanism allows adaptive feature fusion at the sample level, improving robustness under unseen noise distributions.

These results validate the effectiveness of the proposed framework, particularly in challenging scenarios involving severe noise and distribution mismatch.

### 4.4. Ablation Study

To systematically evaluate the contribution of each component in the proposed framework, ablation experiments are conducted by progressively adding different modules. Specifically, four variants are considered: (1) the baseline WDCNN, (2) Improved_WDCNN with enhanced backbone, (3) DUF-Net with dual-path structure and fixed fusion, and (4) the full RGAF-Net with reliability-guided routing.

#### 4.4.1. Ablation Results on PU Dataset

The ablation results on the PU dataset are presented in Table 6. From the results, a clear progressive improvement can be observed.

First, the enhanced backbone (Improved_WDCNN) significantly improves performance over the original WDCNN, demonstrating that multi-scale feature extraction and deeper representation learning effectively enhance robustness under moderate noise conditions.

Second, introducing the dual-path structure (Dual_Uniform) leads to further gains, indicating that separating stable semantic features and adaptive-path structural features provides complementary information even with a fixed fusion strategy.

Finally, incorporating the reliability-guided routing mechanism (RGAF-Net) achieves the best performance. Although the improvement over Dual_Uniform is relatively moderate, it reflects the effectiveness of adaptive feature fusion in refining the learned representations.

#### 4.4.2. Ablation Results Under Severe Noise (JNU Dataset)

To further evaluate the effectiveness of each component under more challenging conditions, additional ablation experiments are conducted on the JNU dataset with severe noise (−10 dB), as shown in Table 7.

Compared with the PU dataset, the JNU task involves significantly stronger noise interference, making it more suitable for evaluating model robustness under extreme conditions.

It can be observed that the performance gap between different variants becomes more pronounced under this setting. The enhanced backbone again contributes the largest improvement, confirming its critical role in feature representation under severe noise.

The dual-path structure further improves performance, demonstrating its ability to capture complementary features even when the signal is heavily corrupted.

More importantly, the reliability-guided routing mechanism consistently brings additional improvements. Compared with DUF-Net, the proposed RGAF-Net introduces only a minor increase in model parameters while achieving consistently improved performance under severe cross-noise conditions. This indicates that the performance gain is not merely caused by increased model capacity, but also benefits from the proposed reliability-guided adaptive fusion mechanism.

#### 4.4.3. Discussion

Overall, the ablation results on both PU and JNU datasets reveal a consistent hierarchical contribution of different components. The enhanced backbone provides the primary improvement in representation capability, while the dual-path structure introduces complementary feature modeling. On top of this, the reliability-guided routing mechanism acts as a refinement module that enhances the adaptability of feature fusion. Its contribution is more evident under severe noise conditions, where sample-wise variability is more significant.

These results demonstrate that the proposed framework achieves a balance between strong feature representation and adaptive capability, leading to improved robustness across different noise levels.

The comparison between DUF-Net and RGAF-Net under the same dual-path backbone architecture that Although RGAF-Net introduces additional routing and reliability estimation modules, the parameter increase compared with DUF-Net is relatively minor, since most parameters are contributed by the shared dual-path backbone architecture.The observed performance improvement therefore mainly reflects the effectiveness of adaptive feature fusion rather than a simple increase in model capacity.

### 4.5. Confusion Matrix and Feature Visualization Analysis

To further evaluate the discriminative capability and feature representation quality of the proposed method under severe noise conditions, confusion matrices and t-SNE visualizations are provided for qualitative analysis. All results are obtained on the PU dataset under the target noise condition of −4 dB.

#### 4.5.1. Confusion Matrix Analysis

**Figure 9:**
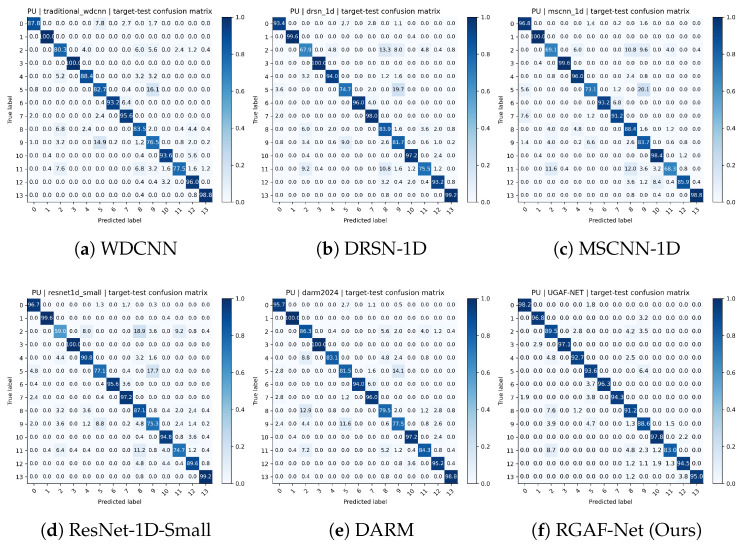
Confusion matrices of different methods on the PU dataset under severe noise condition (−4 dB).

The confusion matrices of all compared methods are shown in Figure 9.

From Figure 9, it can be observed that baseline methods such as WDCNN exhibit significant misclassification across multiple categories. The predictions are relatively dispersed, indicating that the extracted features lack sufficient discriminative power under strong noise interference.

Methods with enhanced architectures, such as DRSN-1D and MSCNN-1D, show improved classification stability, with clearer diagonal patterns compared to WDCNN. However, confusion between certain fault categories still exists, especially for classes with similar fault characteristics.

In contrast, the proposed RGAF-Net demonstrates a more concentrated diagonal distribution, indicating a higher proportion of correctly classified samples. The off-diagonal confusion regions are noticeably reduced, suggesting that the model is capable of maintaining more stable class boundaries even under severe noise conditions.

#### 4.5.2. t-SNE Visualization Analysis

**Figure 10:**
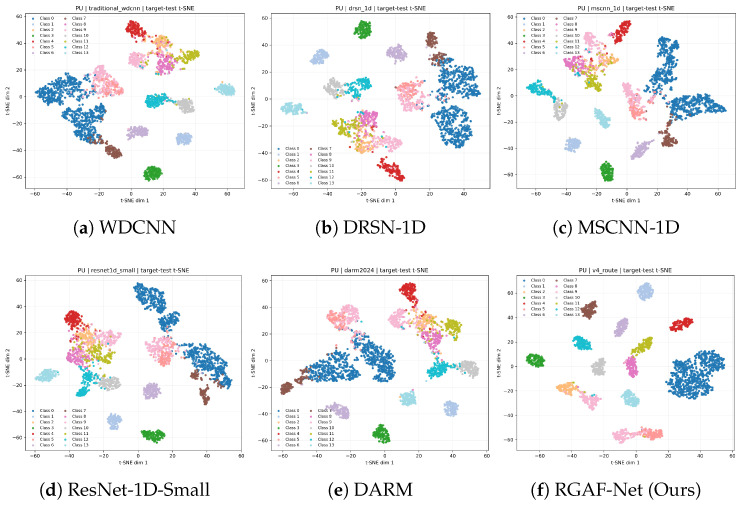
t-SNE visualization of learned feature embeddings on the PU dataset under severe noise condition (−4 dB).

To further analyze the discriminative capability of learned feature representations, t-SNE visualization is adopted to intuitively compare feature distribution of different models, as illustrated in Figure 10. Note that the labels 0–13 in the t-SNE figure correspond to the 14 fault types in the dataset (i.e., label 0 corresponds to fault type 1, label 1 corresponds to fault type 2, …, label 13 corresponds to fault type 14).

As observed in Figure 10, distinct differences in feature distribution patterns can be clearly observed among competing models.

Traditional baseline methods including WDCNN and DARM suffer from severe inter-class cluster overlap, which reveals weak feature discriminability in the latent space. This phenomenon well accounts for their unsatisfactory classification results in quantitative evaluation.

By contrast, comparative models such as MSCNN-1D and ResNet-1D-Small achieve clearer category separation to some extent. Nevertheless, evident overlaps still exist between partial fault categories, demonstrating limited noise robustness against severe interference.

Remarkably, the proposed RGAF-Net yields much more compact and well-isolated feature clusters. Samples within the same category are tightly aggregated, while explicit decision boundaries are formed between different fault classes. Such visualization results demonstrate that our method can learn highly compact and discriminative feature embeddings, maintaining superior intra-class aggregation and inter-class separability even under extreme noisy interference.

#### 4.5.3. Discussion

The visualization results are consistent with the quantitative performance improvements observed in previous sections.

On the one hand, the enhanced backbone contributes to stronger feature extraction capability, leading to improved cluster compactness. On the other hand, the dual-path structure provides complementary representations as reflected by their distinct activation patterns and feature distributions which helps preserve both stable semantic information and noise-sensitive details.

More importantly, the reliability-guided routing mechanism enhances feature adaptability by enabling sample-wise dynamic fusion. Instead of relying on a fixed representation, the model adjusts feature contributions according to input conditions, resulting in more robust and separable feature distributions.

Overall, these results demonstrate that the proposed method not only improves classification accuracy but also significantly enhances the quality and robustness of learned feature representations under challenging cross-noise scenarios.

## 5. Conclusions

This paper investigates the problem of fault diagnosis under mismatched noise conditions and proposes a reliability-guided adaptive feature fusion framework for cross-noise fault diagnosis.

Specifically, an enhanced WDCNN backbone is first constructed to improve multi-scale feature extraction capability under noisy environments. On this basis, a dual-path feature learning architecture is introduced to provide complementary globally stable structural representations and detail-sensitive local structural responses through different pooling and aggregation strategies.

Furthermore, a lightweight reliability estimation branch is designed to characterize degradation-aware reliability tendencies of input samples. Based on the estimated reliability descriptors, a sample-wise adaptive routing mechanism is employed to dynamically balance dual-path feature fusion under heterogeneous noise conditions.

Experimental results on both the PU and JNU datasets demonstrate that the proposed method achieves consistently improved performance compared with representative fault diagnosis approaches under cross-noise settings. In particular, under severe noise conditions, the proposed framework maintains relatively stable classification performance and lower false alarm rates, indicating improved robustness against noise distribution mismatch.

In addition, ablation studies verify the effectiveness of the enhanced backbone, dual-path feature learning strategy, and reliability-guided adaptive routing mechanism. Visualization analyses further demonstrate improved feature compactness and inter-class separability under severe noise contamination.

It should be noted that the primary performance improvement mainly originates from the enhanced feature extraction capability and complementary dual-path representation learning strategy, while the routing mechanism further improves adaptive feature balancing under heterogeneous degradation conditions.

Future work will focus on more effective reliability modeling strategies and more generalizable adaptive feature fusion mechanisms for broader industrial fault diagnosis applications.

## Figures and Tables

**Table 1:** Definitions of major symbols used in the proposed RGAF-Net framework.

Symbol	Definition
$x\in R^{L}$	Input one-dimensional vibration signal with length *L*
$D_{s},D_{t}$	Source-domain set and unseen target domain under different noise conditions
$H_{s},H_{a}\in R^{C_{1}\times T_{1}}$	Intermediate feature maps after the first pooling stage in the stable path and adaptive path, respectively
$M_{s},M_{a}\in R^{C\times T}$	Final deep feature maps generated by the stable path and adaptive path before global average pooling
$z_{s},z_{a}\in R^{C}$	Global pooled feature representations of the stable path and adaptive path
$\phi \left(x\right)\in R^{m}$	Statistical descriptor vector extracted from the input signal
$q_{i}\in R^{d}$	Reliability descriptor vector of the *i*-th sample
$u_{i}\in \left[0,1\right]$	Scalar reliability-aware degradation indicator of the *i*-th sample
$U\in R^{B\times 1}$	Batch-wise vector form of scalar reliability indicators
$\alpha_{s,i},\alpha_{a,i}\in \left[0,1\right]$	Sample-wise routing coefficients for the stable path and adaptive path
$\alpha_{s},\alpha_{a}\in R^{B\times 1}$	Batch-wise routing coefficient vectors for the two pathways
$f_{i}\in R^{C}$	Final fused feature representation of the *i*-th sample
${\hat{y}}_{i}\in R^{N_{c}}$	Predicted class probability vector of the *i*-th sample
$y_{i}\in R^{N_{c}}$	Ground-truth one-hot label vector of the *i*-th sample
*B*	Mini-batch size
*C*	Feature channel dimension
$N_{c}$	Number of fault categories
$L_{cls}$	Weighted classification loss
$L_{bal}$	Routing balance regularization loss
$L_{ent}$	Routing entropy regularization loss
$\lambda_{1},\lambda_{2}$	Trade-off hyperparameters for different loss terms
ρ	Predefined routing balance factor

**Table 2:** Structural differences and representation tendencies of the two feature pathways.

Component	Stable Path	Adaptive Path
Pooling strategy	Early pooling	Delayed pooling
Feature aggregation	Deep hierarchical aggregation	Local interaction enhanced aggregation
Feature tendency	Globally aggregated structural representations	Local structural responses
Noise behavior	Suppress perturbations	Preserve complementary local details
Representation preference	Robust low-frequency structural patterns	Detail-sensitive transient responses

**Table 3:** Fourteen categories of the PU dataset used in this study. OR and IR denote outer race and inner race faults, respectively; S, R, and M represent single, repetitive, and multiple damages; L1–L3 indicate damage levels; F and P denote fatigue and plastic deformation.

Category	Bearing Code	Damage	Category	Bearing Code	Damage
1	K01–K06	Healthy	8	KB24	(OR + IR) + M + L3 + F
2	KA04	OR + S + L1 + F	9	KB27	(OR + IR) + M + L1 + P
3	KA15	OR + S + L1 + P	10	KI04, KI14	IR + M + L1 + F
4	KA16	OR + R + L2 + F	11	KI16	IR + S + L3 + F
5	KA22	OR + S + L1 + F	12	KI17	IR + R + L1 + F
6	KA30	OR + R + L1 + P	13	KI18	IR + S + L2 + F
7	KB23	(OR + IR) + M + L2 + F	14	KI21	IR + S + L1 + F

**Table 4:** Comparison results on the PU dataset under cross-noise conditions.

Model	Accuracy (%)	Macro-F1 (%)	Macro-FPR
WDCNN	89.27	89.01	0.82
DRSN-1D	91.50	89.57	0.65
MSCNN-1D	91.75	89.80	0.64
ResNet-1D-Small	88.75	85.76	0.87
DARM	91.88	90.86	0.63
**RGAF-Net (ours)**	**95.71**	**93.15**	**0.45**

**Table 5:** Comparison results on the JNU dataset under cross-noise conditions.

Model	Accuracy (%)	Macro-F1 (%)	Macro-FPR
WDCNN	65.12	62.68	3.26
DRSN-1D	71.32	66.72	2.61
MSCNN-1D	81.33	75.42	2.06
ResNet-1D-Small	76.70	68.33	2.71
DARM	66.07	56.41	3.86
**RGAF-Net (ours)**	**84.06**	**81.73**	**1.63**

**Table 6:** Ablation results on the PU dataset.

Model	Accuracy (%)	Macro-F1 (%)	Macro-FPR	Params (×10^3^)
WDCNN	89.27	89.01	0.82	111.2
Improved_WDCNN	92.31	91.24	0.61	327.3
DUF-Net	93.89	92.08	0.51	634.6
RGAF-Net	95.71	93.15	0.45	707.6

**Table 7:** Ablation results on the JNU dataset under −10 dB noise condition.

Model	Accuracy (%)	Macro-F1 (%)	Macro-FPR	Params (×10^3^)
WDCNN	65.12	62.68	3.26	111.2
Improved_WDCNN	82.71	79.24	1.94	327.3
DUF-Net	83.11	80.32	1.79	634.6
RGAF-Net	84.56	81.73	1.63	707.6

## Data Availability

The public datasets used in this study are available at the Public Dataset Repository of the Electrical Machines Laboratory, Paderborn University (PU, https://groups.uni-paderborn.de/kat/BearingDataCenter/, accessed on 22 May 2026).
